# Effects of Intravenous Injection of *Porphyromonas gingivalis* on Rabbit Inflammatory Immune Response and Atherosclerosis

**DOI:** 10.1155/2015/364391

**Published:** 2015-05-03

**Authors:** Gengbing Lin, Shuai Chen, Lang Lei, Xiaoqing You, Min Huang, Lan Luo, Yanfen Li, Xin Zhao, Fuhua Yan

**Affiliations:** ^1^School and Hospital of Stomatology, Fujian Medical University, and Stomatological Key Laboratory of Fujian College and University, Fuzhou 350000, China; ^2^Nanjing Stomatological Hospital, Medical School, Nanjing University, Nanjing 210000, China

## Abstract

The effects of intravenous injection of *Porphyromonas gingivalis* (Pg) on rabbit inflammatory immune response and atherosclerosis were evaluated by establishing a microamount Pg bacteremia model combined with high-fat diet. Twenty-four New Zealand rabbits were randomly divided into Groups A-D (*n* = 6). After 14 weeks, levels of inflammatory factors (C-reactive protein (CRP), tumor necrosis factor-*α* (TNF-*α*), interleukin-6 (IL-6), and monocyte chemoattractant protein-1 (MCP-1)) in peripheral blood were detected by ELISA. The aorta was subjected to HE staining. Local aortic expressions of toll-like receptor-2 (TLR-2), TLR-4, TNF-*α*, CRP, IL-6, matrix metallopeptidase-9, and MCP-1 were detected by real-time PCR, and those of nuclear factor-*κ*B (NF-*κ*B) p65, phospho-p38 mitogen-activated protein kinase (MAPK), and phospho-c-Jun N-terminal kinase (JNK) proteins were detected by Western blot. Intravenous injection of Pg to the bloodstream alone induced atherosclerotic changes and significantly increased systemic and local aortic expressions of inflammatory factors, NF-*κ*B p65, phospho-p38-MAPK, and JNK, especially in Group D. Injection of microamount Pg induced inflammatory immune response and accelerated atherosclerosis, in which the NF-*κ*B p65, p38-MAPK, and JNK signaling pathways played important roles. Intravenous injection of Pg is not the same as Pg from human periodontitis entering the blood stream. Therefore, our results cannot be extrapolated to human periodontitis.

## 1. Introduction

Epidemiological studies have associated periodontitis with atherosclerosis [1–3], and a multivariate analysis showed that periodontal pathogen load was positively correlated with the incidence rate of cardiovascular disease [4]. Pathogenic bacteria and products in the periodontal pocket can invade systemic circulation through inflammation-injured epithelial structure [5, 6].* Porphyromonas gingivalis* (Pg), as one of the main pathogenic bacteria for periodontitis, expresses significantly more specific IgG in the serum of patients with severe periodontitis [7]. Besides, Pg DNA was once detected in atherosclerotic plaque tissues [8], and Pg promoted the onset of early atherosclerosis in apolipoprotein E-deficient mice through oral infection [7]. Therefore, Pg participates in the onset of atherosclerosis by invading the circulatory system through periodontal tissues.

In the current study, asymptomatic bacteremia was induced several times by intravenous injection with low-concentration Pg suspension to observe inflammatory response and progression of atherosclerosis. The expressions of molecules in inflammation-related signaling pathways were detected to clarify the relationship between periodontitis and atherosclerosis.

## 2. Materials and Methods

### 2.1. Experimental Animals

Adult male New Zealand rabbits weighing (2.5 ± 0.5) kg were with complete permanent dentition, integral teeth, and healthy periodontal structures, purchased from Shanghai Laboratory Animal Center, Chinese Academy of Sciences [License: SCXK (Shanghai) 2007-0007], and were fed in Department of Comparative Medicine, Fuzhou General Hospital of Nanjing Military Region. Under aseptic conditions, the rabbits were fed in individual cages with the dark/light cycle of 12 h/12 h at 19–29°C, with free access to drinking and eating. This study has been approved by the Institutional Animal Care and Use Committee of Fujian Medical University and was performed according to the ARRIVE (Animal Research: Reporting in Vivo Experiments) guideline.

### 2.2. Experimental Strain

Pg was cultured overnight under anaerobic conditions in culture medium containing 37 g/L bovine brain-heart infusion broth, 5 g/L yeast extract, 5 mg/L hemin, and 0.2 mg/L menadione.

### 2.3. Main Reagents

Ketamine hydrochloride injection was purchased from Fujian Gutian Pharmaceutical Co., Ltd. (China). Bovine brain-heart infusion broth was bought from OXIOD (UK). BASO rapid Gram stain was obtained from Zhuhai Beisuo Biological Technology Co., Ltd. (China). Rb antinuclear factor-*κ*B (NF-*κ*B) p65, Rb antiphospho-c-Jun N-terminal kinase (JNK), and Rb antiphospho-p38 mitogen-activated protein kinase (MAPK) were purchased from Bio Basic Inc. Mouse-anti-rabbit HRP was bought from Jackson. ELISA kit was obtained from Uscn Life Science, Inc. (China). Real-time PCR reagent was purchased from Invitrogen Life Technologies (USA).

### 2.4. Main Apparatus

The main apparatus included EQU307 small transfer electrophoresis tanks (BBI, USA), electrophoresis system (Bio-Rad, USA), qPCR system (Applied Biosystems7500 Fast, USA), AV2700 Automatic Biochemical Analyzer (Olympus, Japan), High-speed refrigerated centrifuge (Heraeus, Germany), ultracentrifuge (Hitachi, Japan), tissue paraffin slicer (Thermo, USA), automated enzyme immunoassay analyzer (Human, Germany), ultra-pure water system (Millipore, France), and AnaeroPack anaerobic culture equipment (Mitsubishi, Japan).

### 2.5. Experimental Procedure

**Figure 1:**
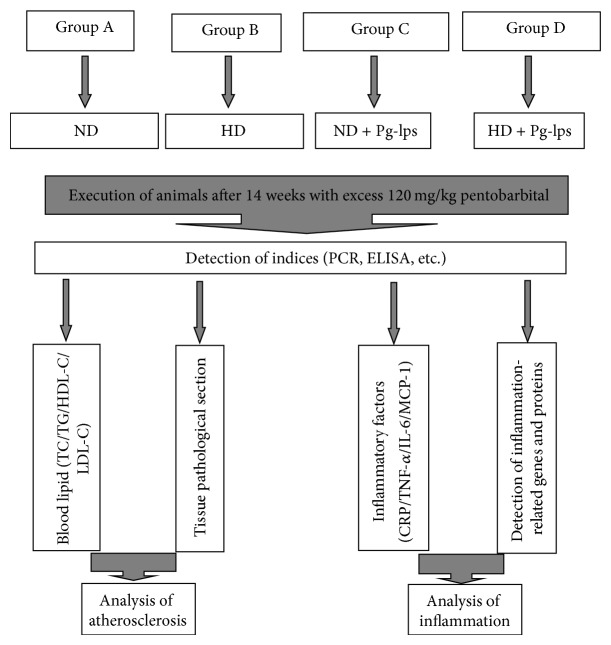
Experimental procedure. ND: normal diet; HD: high-fat diet.

The experimental procedure is shown in Figure 1.

#### 2.5.1. Animal Grouping

After at least two weeks of adaptive feeding, 24 New Zealand rabbits were randomly divided into four groups (*n* = 6).

#### 2.5.2. Establishment of High-Fat Diet Animal Model

For 14 consecutive weeks, Group A and Group C were given normal diet (150–200 g), and Group B and Group D were fed with high-fat diet (150–200 g), all with free access to water. The high-fat diet consisted of basic feed, 15% fresh egg yolk, 1% cholesterol, and 5% lard.

#### 2.5.3. Establishment of Chronic Subclinical Pg Bacteremia Model

Pg was passaged in contamination-free single colonies, centrifuged, and prepared into a 1.0 × 10^8^ CFU/mL suspension with PBS by using McFarland standard tubes as the reference.

After six weeks of normal or high-fat diet feeding, Group A and Group B were injected with PBS (0.1 mL/kg), while Group C and Group D were injected with Pg suspension into the marginal ear vein (10^8^ CFU, 0.1 mL/kg). The injection was performed three times per week for eight consecutive weeks [9]. Group C and Group D were also subjected to periodontal ligature.

#### 2.5.4. Execution of Experimental Animals

After 14 weeks, the rabbits were euthanized by injecting 120 mg/kg ketamine hydrochloride.

### 2.6. Blood Examination

Blood (10 mL) was collected after 12 h of fasting from the central ear artery on the 1st day of experiment and before execution and centrifuged at 3000 g for 5 min, from which the supernatant was obtained. Levels of interleukin-6 (IL-6), tumor necrosis factor-*α* (TNF-*α*), C-reactive protein (CRP), and monocyte chemoattractant protein-1 (MCP-1) in the peripheral blood were determined by ELISA kits, and those of total cholesterol (TC), triglyceride (TG), low-density lipoprotein-cholesterol (LDL-C), and high-density lipoprotein-cholesterol (HDL-C) were measured by OLYMPUS AV2700 Automatic Biochemical Analyzer.

### 2.7. Aortic Morphology and Detection of Inflammation-Related Indices

#### 2.7.1. Preparation of Tissue Sections

About 1.5 cm of the proximal aortic segment was collected and rinsed roughly with normal saline. Ultrathin sections (4 *μ*m) were prepared and dried overnight in a 60°C oven. Then they were deparaffinized in turpentine I and turpentine II for 10 min and hydrated in absolute ethanol I for 10 min, in absolute ethanol II for 5 min, in 95% ethanol for 5 min, in 80% ethanol for 5 min, and in 70% ethanol for 5 min. After cleaning, the sections were stained by hematoxylin for 10 min, washed by running water for 10 min, stained by eosin for 20 s, and dehydrated by 95% ethanol I, 95% ethanol II, absolute ethanol I, and absolute ethanol II sequentially (2 min each). The sections were thereafter rendered transparent by using xylene and sealed with neutral gum.

#### 2.7.2. Observation and Quantitative Morphological Analysis

Three sections of the aorta separated by 80 *μ*m were subjected to HE staining. With 100x magnification, four visual fields were selected for each section to determine the thicknesses of tunica intima and tunica media and their ratios. The average of three replicates was finally used.

Within 10 min after the rabbits died, aortic tissues not thicker than 0.5 cm were sampled after blood stains and contaminants were removed. They were then dried with sterile gauze and rapidly stored in liquid nitrogen. Levels of TLR-2, TLR-4, MCP-1, TNF-*α*, IL-6, CRP, and MMP-9 were detected by real-time PCR, and those of NF-*κ*B p65, phospho-p38-MAPK, and phospho-JNK were detected by Western blotting.

#### 2.7.3. Real-Time PCR

In liquid nitrogen, 10 mg aortic tissues were homogenized, put in 1.5 mL centrifuge tubes, added 1000 *μ*L of Trizol, and left still for 5 min. After addition of chloroform, the tubes were votexed for 10 s, left still for 5 min, and centrifuged at 12000 g and 4°C for 15 min, from which the supernatant was transferred into a 1.5 mL centrifuge tube, added to equal volume of isopropanol, shaken, and left still at −20°C for 1 h and at room temperature for 10 min. After the solution was centrifuged at 12000 g and 4°C for 10 min, the supernatant was discarded and isopropanol was removed, into which 1 mL of absolution ethanol was added. After another centrifugation at 12000 g and 4°C for 5 min, the supernatant was discarded and diethylpyrocarbonate- (DEPC-) treated water was added when the precipitate was dry. The product was stored at −80°C prior to use.

Mix 1 was obtained by adding the following compounds in a sterile RNase-free Eppendorf tube: 5 *μ*L of DEPC-treated water, 1 *μ*L of 10 mM dNTP Mix, 0.5 *μ*L of random primer, 0.5 *μ*L of primer (50 *μ*M oligio(dt)), and 5 *μ*L of up to 5 *μ*g total RNA, 12 *μ*L in total; then mix 1 was heated at 65°C for 5 min and cooled on ice for 1 min, and mix 2 was prepared by adding the following compounds: 12 *μ*L of Mix 1, 1 *μ*L of SuperScrip III RT (200 U/*μ*L), 2 *μ*L of 0.1 M dTT, 1 *μ*L of 40 U/*μ*L RNaseout, and 4 *μ*L of 5x first-strand buffer, 20 *μ*L in total; after reaction at 25°C for 5 min, 50°C for 60 min, and 70°C for 15 min, the cDNA product was cooled on ice immediately and stored at −20°C prior to use.

PCR system consisted of 12.3 *μ*L of ultrapure water, 0.2 *μ*L of Taq polymerase (5 U/*μ*L), 0.5 *μ*L of downstream primer (10 *μ*M), 2 *μ*L of Mg^2+^ (25 mM), 1 *μ*L of SYBR (20x), 0.5 *μ*L of upstream primer (10 *μ*M), 0.5 *μ*L of dNTPs (25 mM), 2 *μ*L of 10x PCR buffer, and 1.0 *μ*L of template, 20 *μ*L in total. PCR was performed at 95°C for 2 min and cycled in 40 repeats: 95°C for 10 s, 60°C for 30 s, 70°C for 45 s, and melting at 70–95°C.

Total-length cDNA sequences of TNF-*α*, CRP, IL-6, and MCP-1 were obtained from http://www.ensemble.org/index.html, based on which specific primers for real-time PCR were synthesized (Table 1).

After real-time PCR of target and housekeeping genes of each sample, their levels were directly generated by the analysis system, providing a standard curve simultaneously. Relative expression level *F* = 2^−ΔΔct^, where −ΔΔct = (average ct of target gene − average ct of housekeeping gene)_sample_  − (average ct of target gene − average ct of housekeeping gene)_reference_.

#### 2.7.4. Western Blotting

Aortic tissues were rinsed twice to three times with cold TBS, added to 10 equiv. extractant (phosphorylated protease inhibitors cocktail and PMSF were added several minutes before), homogenized on ice, transferred in a centrifuge tube, shaken, put on ice for 30 min, and centrifuged at 12000 g for 5 min, from which the supernatant was collected as the total protein solution.

Sample protein levels were determined with the Bradford method by plotting a standard curve and measuring the absorbance at 595 nm after adding 900 *μ*L of Bradford's reagent into 1 *μ*L of protein and 99 *μ*L of 0.9% normal saline.

Protein sample (40 *μ*g) was then loaded for Western blotting and transferred to a PVDF membrane that was subsequently blocked in 5% skimmed milk prepared with 0.5% TBST. Afterwards, the membrane was incubated overnight with diluted primary antibodies (5% skimmed milk dissolved with TBST) at 4°C, washed three times with TBST (5 min each time), incubated with TBST-diluted secondary antibodies (1 : 3000) at room temperature for 30 min, washed three times again (5 min each), and subjected to chemiluminescent detection, developing, and fixing. After gel imaging and scanning, the optical densities of target bands were detected by Alpha software.

### 2.8. Statistical Analysis

All data were analyzed by SPSS 19.0. All indices were subjected to normality test, and those conforming to normal distribution were expressed as mean ± standard deviation. Means of multiple groups were compared by one-way analysis of variance, and intergroup comparisons were performed by LSD-*t* test. Pearson's correlation analysis was used. *P* < 0.05 was considered statistically significant.

## 3. Results

### 3.1. Aortic Morphology

**Figure 2:**
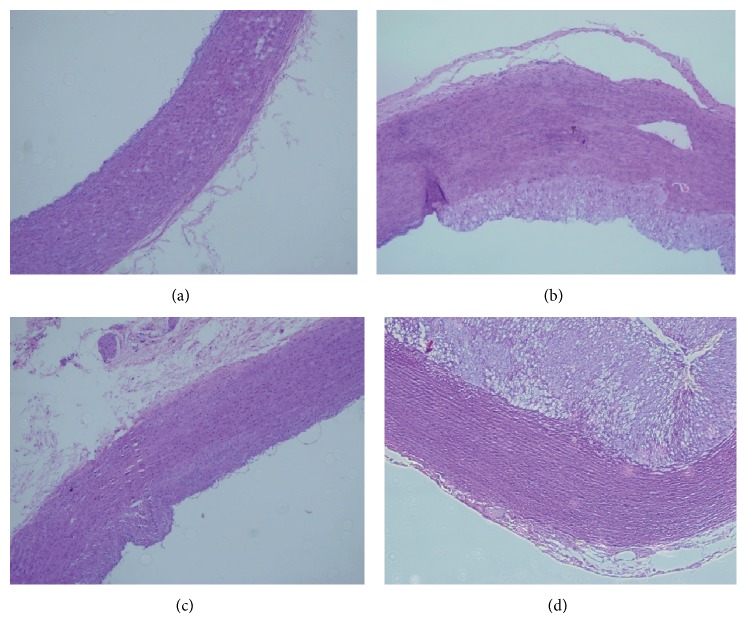
Aortic pathological changes (×100). (a) Group A; (b) Group B; (c) Group C; (d) Group D.

Group A: endothelial cells were intact and adhesive to the internal elastic lamina in single layers, and smooth muscle cells in the tunica media were arranged orderly (Figure 2(a)). Group B: the tunica intima thickened and had edema, and there were 4-5 layers of foam cells and infiltrated inflammatory cells. Meanwhile, the arrangement of elastic fibers in the tunica media was disordered (Figure 2(c)). Group C: there were 1-2 layers of foam cells and infiltrated inflammatory cells, and smooth muscle cells were arranged relatively orderly (Figure 2(b)). Group D: the tunica intima experienced more evident thickening and edema with 8-9 layers of foam cells. Smooth muscle cells obviously decreased in the tunica media, which were accompanied by disorganized, twisted, and structurally undefined elastic fibers, and there were scattered atherosclerotic plaques (Figure 2(d)). In short, high-fat diet alone presented more severe changes in intima and media.

### 3.2. Quantitative Morphological Analysis

Compared with Group A, Group C had significantly thicker tunica intima and significantly higher tunica intima/tunica media ratio, but their tunica media thicknesses were similar. Compared with Group B, however, the two thicknesses and the ratio of Group D all increased significantly (Table 2).

### 3.3. Expressions of Serum Inflammatory Factors

**Figure 3:**
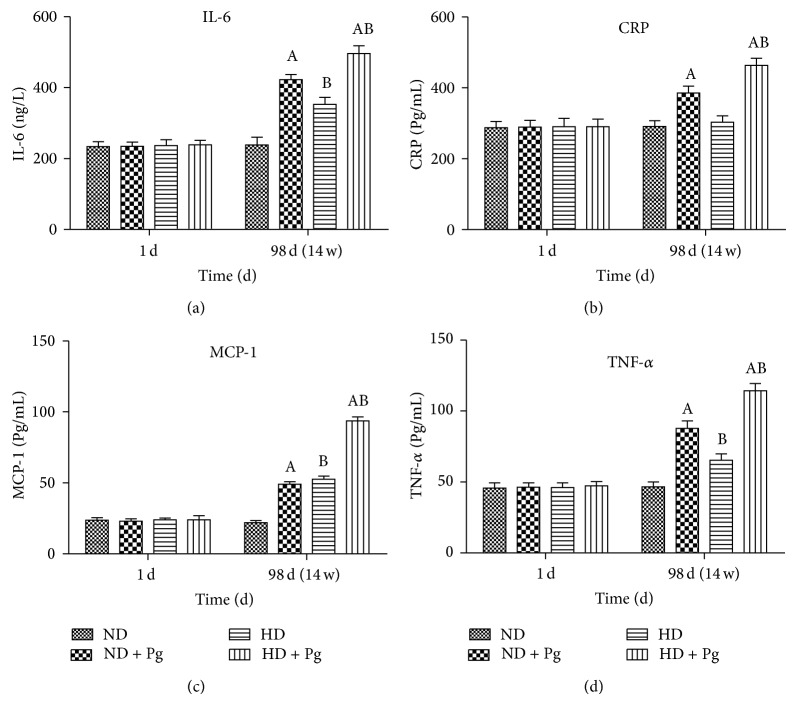
Expressions of inflammatory factors in peripheral blood. A: difference before and after Pg stimulation, *P* < 0.05; B: difference before and after high-fat diet, *P* < 0.05. ND: normal diet; HD: high-fat diet.

The levels of serum CRP, IL-6, TNF-*α*, and MCP-1 of Group C and Group D, especially those of Group D, were significantly higher than those of Group A and Group B, respectively. Compared with Group A, the serum IL-6, TNF-*α*, and MCP-1 levels were significantly higher in the other three groups, but their CRP levels were similar (Figure 3).

### 3.4. Real-Time PCR Detection Results

**Figure 4:**
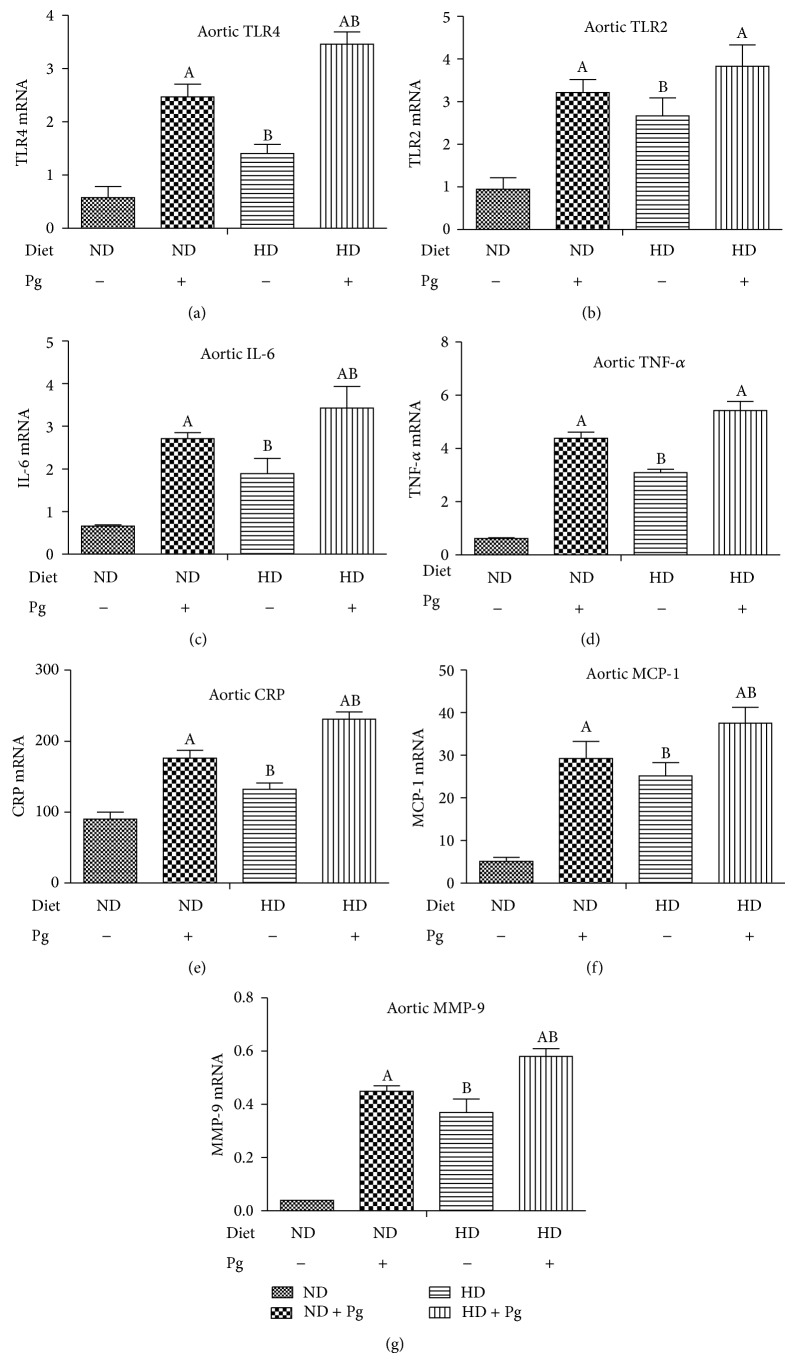
Local aortic expressions of inflammatory factors. A: difference before and after Pg stimulation, *P* < 0.05; B: difference before and after high-fat diet, *P* < 0.05. ND: normal diet; HD: high-fat diet.

Groups B–D had significantly higher expression levels of TLR-2, TLR-4, TNF-*α*, CRP, IL-6, MMP-9, and MCP-1, following a descending order of Group D > Group C > Group B and with significant intergroup differences (Figure 4).

### 3.5. Blood Lipid Detection Results

The TCHO and LDL-C levels in Group C significantly exceeded those in the Group A, whereas the HDL-C level was significantly lower. The two high-fat diet groups had significantly higher TG, TCHO, LDL-C, and HDL-C levels than those of the two normal diet groups. The TCHO and LDL-C levels in Group D were also significantly higher than those in Group B, but the HDL-C level was significantly lower (Table 3).

### 3.6. Western Blotting Results of the NF-*κ*B p65 Pathway

**Figure 5:**
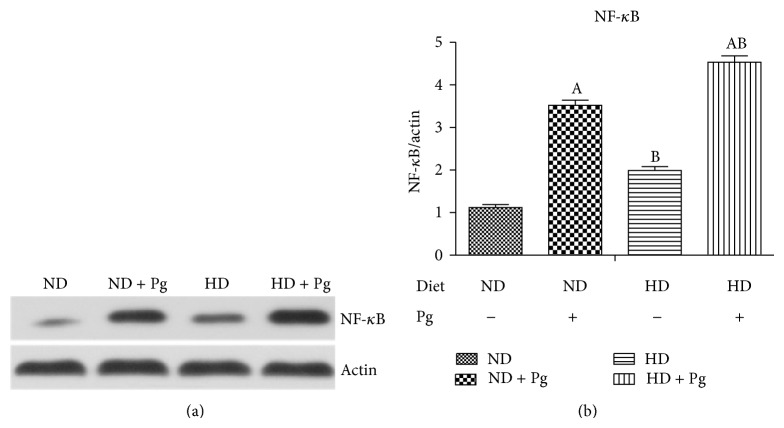
Western blotting results of NF-*κ*B p65 (a) and expression levels (b). A, difference before and after Pg stimulation, *P* < 0.05; B, difference between normal and high-fat diets, *P* < 0.05. ND: normal diet; HD: high-fat diet.

The levels of NF-*κ*B p65 in Groups B–D were significantly higher than that of Group A, following a descending order of Group D > Group C > Group B and with significant intergroup differences (*P* < 0.05) (Figure 5).

### 3.7. Western Blotting Results of the p38-MAPK Pathway

**Figure 6:**
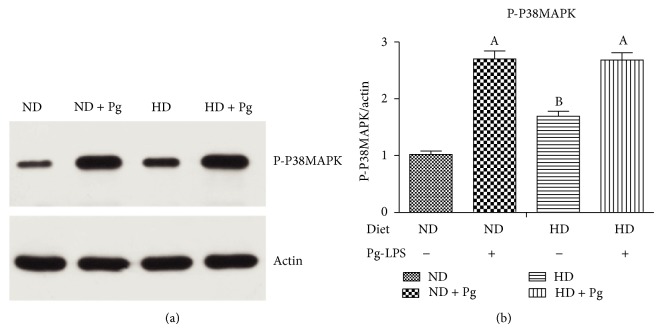
Western blotting results of phospho-p38-MAPK (a) and expression levels (b). A, difference before and after Pg stimulation, *P* < 0.05. ND: normal diet; HD: high-fat diet.

Group C and Group D had significantly higher levels of phospho-p38-MAPK than those of Group A and Group B, respectively. Meanwhile, there were no differences between Group C and Group D or between Group A and Group B (Figure 6).

### 3.8. Western Blotting Results of the JNK Pathway

**Figure 7:**
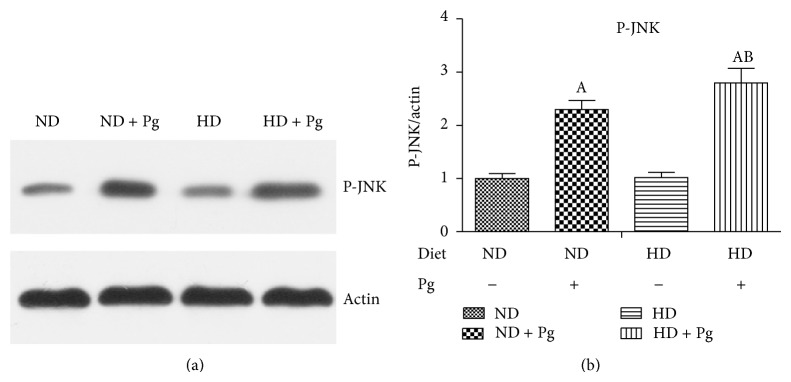
Western blotting results of phospho-JNK (a) and expression levels (b). A, difference before and after Pg stimulation, *P* < 0.05; B, difference between normal and high-fat diets, *P* < 0.05. ND: normal diet; HD: high-fat diet.

Group C and Group D had significantly higher levels of phospho-JNK than those of Group A and Group B, respectively, and the increase in Group D was most evident. There were no differences between Group A and Group B (Figure 7).

## 4. Discussion

Atherosclerosis is a chronic inflammatory disease in which various inflammatory cells and factors are involved [10]. Bacterial infections promote the onset and progression of atherosclerosis by injuring the vascular endothelium and by activating systemic and local inflammatory immune responses [11, 12]. However, the randomized trials to mitigate the inflammatory contribution of these infections (*Chlamydia* and* Helicobacter*) did not reduce atherosclerotic outcomes suggesting potential confounding [13]. On the other hand, metabolic inflammation is one of the main causes of coronary heart disease [14], and the link between oral bacteria and metabolic inflammation has attracted much attention in the research community [15].

Periodontitis has been closely associated with atherosclerosis [16] because the former allows periodontal pathogenic bacteria to enter blood circulation and finally the cardiovascular system through exposed areas by local gingival epithelial inflammation, ulceration, chewing, tooth-brushing, periodontal treatment, and surgery [17–19]. By inducing a continuous, mild systemic inflammatory response, Pg invades the circulatory system through periodontal tissues.

Since Group C suffered from atherosclerotic changes, Pg injection alone was enough to initiate atherosclerosis. In addition, the expressions of inflammatory factors increased in peripheral blood and local aorta. Due to limited experimental conditions, the environment of human periodontitis was not fully simulated. For instance, intravenous injection of Pg did not resemble Pg from human periodontitis entering the blood stream. The results herein suggested that injection of microamount Pg may induce systemic and local aortic inflammatory immune responses, probably being associated with the progression of atherosclerosis. However, injection of Pg is not the same as the condition where human periodontitis affects the pathogenesis of atherosclerosis. Therefore, we cannot translate our results as the simulation of human periodontitis and atherosclerosis relationship.

Groups B–D all underwent atherosclerotic changes, among which the symptom of Group D was most apparent, suggesting that Pg injection in combination with hyperlipidemia accelerated the progression of atherosclerosis. High-fat diet easily accumulates lipids that aggregate macrophages and secret inflammatory cytokines, thus damaging adjacent tissues and organs to trigger a cascade reaction by secreting more inflammatory factors [20–22]. In Group B, the expression levels of TG, TCHO, LDL-C, and HDL-C all significantly increased, and those of most inflammatory factors in the peripheral blood and local aorta were also significantly elevated. Therefore, it is reasonable to postulate that individual high-fat diet sufficed to induce lipid metabolic disorder, and the resulting lipid accumulation results in chronic inflammation. LDL can stimulate monocytes/macrophages to significantly induce the expressions of inflammatory factors and can enhance the proinflammatory activity of monocytes [23]. Hence, high-fat diet induced more obvious inflammatory response than normal diet did under identical stimulation with Pg.

TLRs, especially TLR-4 and TLR-2, are widely expressed in the arteries of atherosclerotic patients [24], and the two receptors are related with Pg and the mechanism of its toxic effects. Pg can also upregulate the expressions of TLR-2, TLR-3, TLR-4, TLR-6, and TLR-9 in vascular endothelial cells and affect the functions of these cells [25]. When TLR-2 expression is blocked, monocytes no longer readily adhere to vascular endothelial cells due to weakened stimulating effects of Pg-LPS and pili [26, 27]. Moreover, Pg-LPS produces inflammatory cytokines by activating vascular endothelial cells through TLR-2 [28]. Macrophages play crucial, versatile roles in the onset and progression of atherosclerosis [29]. Zhou et al. reported that Pg-LPS and pili transduced signals mainly through TLR-2, and Pg* per se* did so through TLR-2 and TLR-4 [30]. Since Pg, Pg-LPS, and pili can induce upregulation of TLR-2 on macrophage surface, Pg infection activated macrophages through TLR-2 [31]. Accordingly, TLRs are essential parts of the signaling of Pg infection-accelerated atherosclerosis, which vary depending on the target cells and Pg virulence factors. Pg injection herein significantly increased local aortic expressions of TLR-2 and TLR-4, indicating that Pg stimulated systemic inflammatory immune response through TLR-2 and TLR-4, finally leading to atherosclerosis.

Although there are numerous types of proinflammatory factors for atherosclerosis, only the MAPK and NF-*κ*B signaling pathways are mainly responsible for regulating these factors, the blockage of which may be able to hinder atherosclerotic progression.

Overactivation of the NF-*κ*B pathway that regulates target genes involved in inflammatory response is closely related with the progression of atherosclerosis [32]. In this study, microamount Pg stimulation significantly increased NF-*κ*B, suggesting that the NF-*κ*B pathway predominantly controlled Pg-induced inflammatory immune response and exerted chain amplifying effects on proinflammatory factors for atherosclerosis [33]. Thus, Pg and its virulent factors as well as high-fat diet-induced high-concentration LDL stimulation can induce systemic inflammatory response that activates the NF-*κ*B pathway in artery endothelial cells and subendothelial inflammatory cells, which further increases inflammatory factors and leads to a vicious circle as the pathogenesis of Pg-induced atherosclerosis.

The MAPK pathway mediates extracellular signals to induce nuclear reactions and regulates cytokines, adhesion molecules, and chemokines, critically controlling the regulation of systemic inflammatory response. Besides being widely related with the NF-*κ*B pathway, the JNK and p38-MAPK pathways are also closely associated with inflammatory response. Pg stimulation herein significantly elevated the local aortic expression levels of phospho-p38-MAPK and JNK, so the JNK and p38-MAPK pathways essentially participated in Pg-induced local inflammatory immune response. Similarly, Pg pili can activate the p38-MAPK pathway in human peripheral blood monocytes and release IL-6 to participate in inflammatory response by activating the NF-*κ*B pathway [34]. Moreover, Pg-LPS can activate the p38-MAPK pathway in human monocytes but not in endothelial cells [35]. Furthermore, Pg is able to activate the JNK pathway in gingival epithelial cells [36], which, however, has never been reported to be activated in monocytes/macrophages or vascular endothelial cells. NF-*κ*B pathway, MAPK pathway, and JNK pathway are universal inflammatory pathways. Thus, any biochemical reaction that involves inflammation will activate these pathways.

## 5. Conclusion

In summary, stimulation by injecting microamount Pg into the bloodstream induced inflammatory immune response and further accelerated atherosclerotic progression, on which TLR-2, TLR-4, and the NF-*κ*B p65, p38-MAPK, and JNK signaling pathways exerted bridging effects.

## Figures and Tables

**Table 1:** PCR primer sequences.

Gene	Primer
IL-6-F1	CCTGAGGCCAAAGGTCAAGAA
IL-6-R1	GTGGTCGTCTTCAGCCACTG
MCP-1-F1	GCCCAGGCTGAGCACG
MCP-1-R1	CCCAGCACGACGTTCCC
CRP-F1	GCTGCTGTGGTGTTTCCTGA
CRP-R1	CTTCTTGTGCATGCCTGCCT
TNF-*α*-F1	CCCACGTAGTAGCAAACCCG
TNF-*α*-R1	TTGTCCGTGAGCTTCATGCC

**Table 2:** Thicknesses of tunica intima and tunica media and their ratios (mean ± SD).

Group (*n* = 6)	Id (*μ*m)	Md (*μ*m)	I/M
Group A	21.74 ± 8.05	129.93 ± 17.01	0.16 ± 0.03
Group B	237.12 ± 13.77^#^	202.33 ± 21.34^#^	1.17 ± 0.63^#^
Group C	83.15 ± 6.76^∗^	132.09 ± 38.37	0.25 ± 0.05^∗^
Group D	377.32 ± 11.57^&^	252.37 ± 26.37^&^	1.49 ± 0.85^&^
*F* value	24.341	19.541	17.753
*P* value	<0.01	<0.01	<0.01

^∗^Significant difference compared with Group A; ^#^significant difference compared with Groups A and C; ^&^significant difference compared with Groups A–C.

**Table 3:** Expressions of blood lipid indices (*x* ± *s*, mmol/L).

Group	TG	TCHO	LDL-C	HDL-C
Group A	0.78 ± 0.21	1.28 ± 0.12	0.27 ± 0.03	0.64 ± 0.14
Group B	3.94 ± 1.36^#^	16.67 ± 7.90^#^	15.01 ± 9.04^#^	2.06 ± 0.07^#^
Group C	0.72 ± 0.52	8.37 ± 1.67^∗^	9.51 ± 1.39^∗^	0.39 ± 0.21^∗^
Group D	4.13 ± 0.81^#^	25.07 ± 5.27^&^	26.37 ± 7.21^&^	1.02 ± 0.31^&^
*F* value	21.125	19.522	22.312	14.745
*P* value	<0.01	<0.01	<0.01	<0.05

^∗^Significant difference compared with Group A; ^#^significant difference compared with Groups A and C; ^&^significant difference compared with Groups A–C.
